# ﻿Lectotypification of two names of *Carexbuekii* hybrids (Cyperaceae) and notes on their morphology, ecology and distribution

**DOI:** 10.3897/phytokeys.236.113435

**Published:** 2023-12-21

**Authors:** Helena Więcław, Radomír Řepka, Jacob Koopman

**Affiliations:** 1 Institute of Marine and Environmental Sciences, University of Szczecin, Adama Mickiewicza 18, 70-383, Szczecin, Poland University of Szczecin Szczecin Poland; 2 Department of Forest Botany, Dendrology and Geobiocenology, Faculty of Forestry and Wood Technology, Mendel University, Zemědělská 3, CZ-613 00 Brno, Czech Republic Mendel University Brno Czech Republic; 3 ul. Kochanowskiego 27, 73-200 Choszczno, Poland Unaffiliated Choszczno Poland

**Keywords:** *Carex* ×*ligniciensis*, *Carex* ×*vratislaviensis*, section *Phacocystis*, typification, WRSL herbarium

## Abstract

Lectotypes are designated for two *Carexbuekii* hybrid names. The typification is supplemented with notes on their morphology, ecology, and distribution.

## ﻿Introduction

*Carex* L. (Cyperaceae) is one of the most species-rich angiosperm genera with more than 2,000 species distributed worldwide (POWO 2023). In a large genus like *Carex* hybridisation is especially frequent, however most of the *Carex* hybrids are restricted to a few sections, e.g. *Ceratocystis* Dumort., *Glareosae* G.Don, *Phacocystis* Dumort., and *Vesicariae* Heuff. (Cayouette and Catling 1992, Wallnöfer 2006, Więcław and Koopman 2013, Pedersen et al. 2016).

*Carexbuekii* Wimm. belongs to the section Phacocystis, one of the largest and taxonomically most complex sections within the genus *Carex*, with about 110 species distributed worldwide. Furthermore, hybridisation is frequent in *Phacocystis*, and several species are of hybrid origin (Roalson et al. 2021). *Carexbuekii* has hybridised with four other *Phacocystis* species so far: *C.acuta* L., *C.cespitosa* L., *C.elata* All., and *C.nigra* (L.) Reichard (Koopman 2022), all these four hybrids were described by Figert (1900, 1907).

Ernst Figert (1848–1925), a German (Prussian) teacher and botanist from Liegnitz (nowadays named Legnica, Poland) collected plants mainly from Lower Silesia, paying attention to difficult genera, e.g. *Carex*, *Salix* L., *Mentha* L., and their hybrids. Figert (1900) described the first two hybrids of *C.buekii*, C.×ligniciensis Figert [*C.buekii* × *C.nigra*] and C.×vratislaviensis Figert [*C.acuta* × *C.buekii*], based on plants collected on the same date and at the same site in Silesia (Poland). Figert (1900) did not select a type specimen or provide an illustration for C.×ligniciensis. Neither did he do so for C.×vratislaviensis when he named and described these two hybrids. Original material of C.×ligniciensis and C.×vratislaviens was found in the herbarium of WRSL (Poland). Both sheets were originally labelled by Figert. These hybrids are usually intermediate to the parental species and exhibit a wide range of morphological variability. *Carexbuekii* hybrids, especially C.×vratislaviensis may be fertile and backcrosses appearing in populations are difficult to identify. These issues can lead to nomenclatural and taxonomic confusion and a lack of clarity in limits between parental species and hybrids. The lectotype of *C.buekii* has been designated (Jiménez-Mejías et al. 2014), the next step is the typification of its hybrids.

## ﻿Material and methods

Taxonomic literature, including protologues, as well as fresh collections from parts of the Czech Republic and Poland, were examined. We also examined dried specimens deposited at the herbaria of BRNM, BRNU, JE, PR, PRC, and WSRL (acronyms based on Thiers 2023, continuously updated) and used the online database (JACQ Virtual Herbaria 2023) to check for type specimens. We have designated lectotypes, by comparing specimens with protologues, and selecting the most complete ones, in accordance with Art. 9.3 of the “Shenzhen Code” (Turland et al. 2018).

## ﻿Results and discussion

### ﻿ Carex × ligniciensis

Taxon classificationPlantaePoalesCyperaceae

Figert, Allg. Bot. Z. Syst. 6: 38 (1900) [C. buekii × C. nigra].

E4AAC782-48C2-5493-85DF-19C7838B78A6

#### Lectotype (designated here).

**Figure 1.**
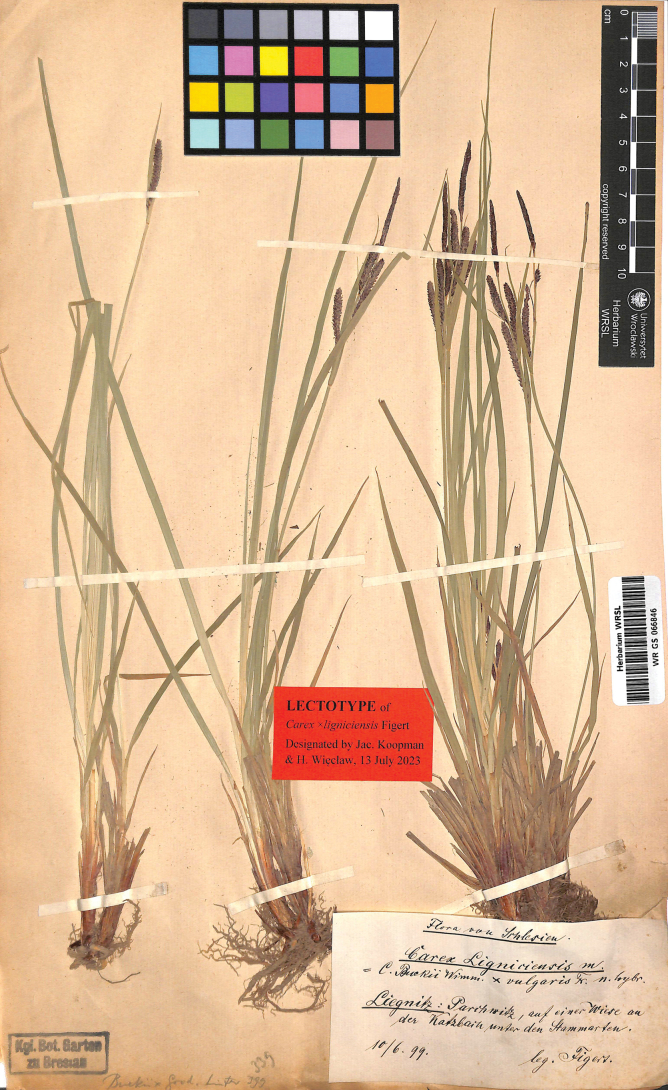
The lectotype of Carex×ligniciensis Figert (WRSL barcode WR GS 066846). Photo: Herbarium, Museum of Natural History University of Wrocław, Poland.

Poland. Flora von Schlesien. Liegnitz: Parchwitz, auf einer Wiese an der Katzbach unter den Stammarten. 10/6/99. Leg. Figert (WRSL barcode WR GS 066846; isolectotype WRSL barcode WR GS 058738) (Fig. 1).

#### Morphology.

The hybrid is mostly intermediate between the parental species and characterised by the following traits: ± tussocks 25–40 cm high, with numerous, shorter or longer creeping rhizomes; stems slender, with reddish brown to purple scale-like, non-reticulate basal sheaths, rough on the edges in the lower half; leaf blades 3–4 mm wide, with very long acuminate, bristle-like tip, very rough on the margin, dark green to grey-green; male spikes 1–2, oblong-cylindrical, glumes brown-black to black, obtuse, with a light central stripe, female spikes 3(–4), narrow, short cylindrical, proximate, lowermost slightly distant, lax at base, ca 4 cm long, pedunculate; female glumes ovate, shorter than utricles, dark brown; utricles empty, small, non-deciduous, green, without veins; lower bract shorter than inflorescence (Grulich et al. 2023). Wallnöfer (2006) stated that this hybrid has amphistomatic leaves (stomata on both sides of the leaves). This trait makes this hybrid impossible to confuse with the other *C.buekii* hybrids, which have only stomata on the lower surface of the leaves (hypostomatic). The first of the parental species, *C.buekii*, is hypostomatic while in the second one, *C.nigra*, the stomata are found on the upper (adaxial) side of the leaves (epistomatic).

#### Ecology.

This hybrid was found in floodplains of large rivers where both parental species could meet. However, *C.nigra* avoids warm areas with the exception of isolated lowland fen sediments in previously flooded meadows, which corresponds to all known finds of this hybrid so far.

#### Distribution.

Carex×ligniciensis is relatively rare and has been found so far in Poland, the Czech Republic and Italy (Koopman 2022). The specimens in BRNL, BRNM, CB, PR, and PRA were collected in the Czech Republic between 1921 and 1995, and they lack field verification. Therefore, we could consider it missing or even extinct at this locality. On the other hand, C.×ligniciensis is a very inconspicuous and apparently overlooked plant. In the Czech Republic, only one recent locality is known from the floodplain of the River Morava near the town of Kroměříž. As far as we know there are no recent findings of this hybrid in Poland, while its occurrence in Italy is at least questionable, as *C.buekii* is extremely rare in this country (Koopman et al. 2018).

The sterility of C.×ligniciensis limits it dispersal, however, the persisting of hybrid populations probably depends on vegetative reproduction, like with other sterile hybrids in *Carex* (Pedersen et al. 2016). The spontaneous recurrence and survival of hybrids under natural conditions are a driving force of plant speciation (e.g. Mallet 2007, Soltis 2013).

### ﻿ Carex × vratislaviensis

Taxon classificationPlantaePoalesCyperaceae

Figert, Allg. Bot. Z. Syst. 6: 39 (1900) [C. acuta × C. buekii].

123BC0B8-DACB-5762-B6B6-50E40712FB81

≡ C.buekiiWimmervar.melanostachya R. Uechtr., Jahresber. Schles. Ges. Vaterl. Cult. 43: 236 (1865, publ. 1866).

#### Lectotype (designated here).

**Figure 2.**
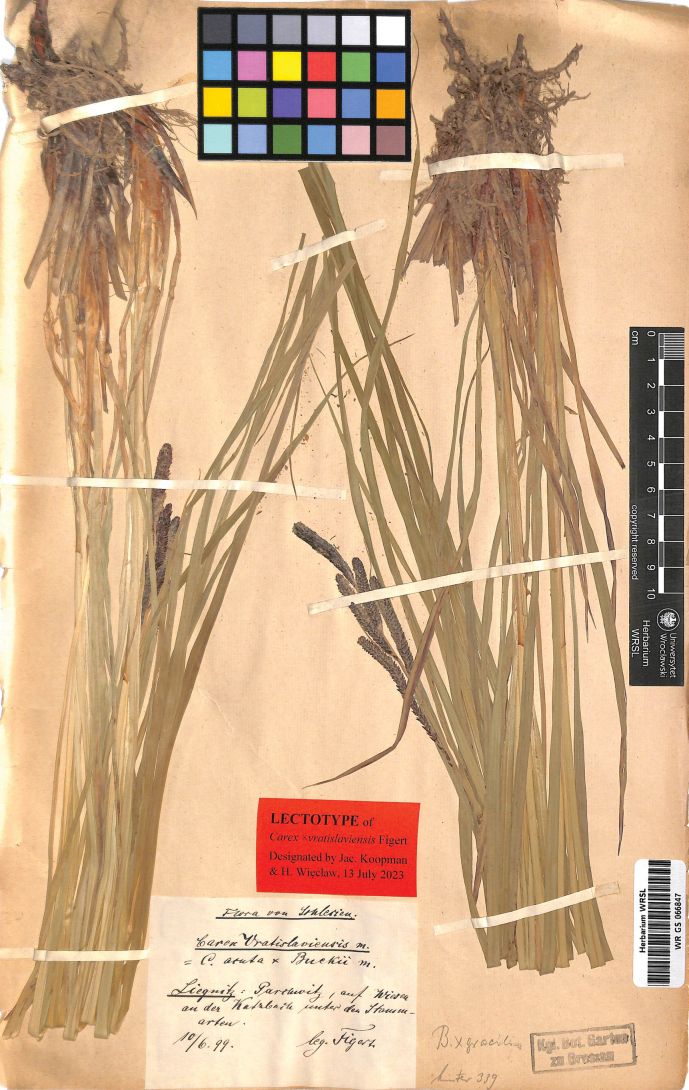
The lectotype of Carex×vratislaviensis Figert (WRSL barcode WR GS 066847). Photo: Herbarium, Museum of Natural History University of Wrocław, Poland.

Poland. Flora von Schlesien. Liegnitz: Parchwitz, auf Wiesen an der Katzbach unter den Stammarten. 10/6/99. Leg. Figert (WRSL barcode WR GS 066847; isolectotypes: WRSL barcode WR GS 058739; JE barcode JE 00021673, barcode JE 00026167, barcode JE 00026168, barcode JE 00026169) (Fig. 2).

#### Morphology.

This hybrid is very variable, often intermediate between the parental species, but also tends to be morphologically closer to one of the parents. The utricles are very different in shape and size, from small ones similar to *C.buekii*, to more often closer in size to *C.acuta*. The leaf sheaths vary with the gene flow of the parental species: from reddish brown, robust, scale-like, shiny, reticulate, to intermediate types with smaller and slender sheaths than *C.buekii*, dark reddish brown, in spring with distinctive reticulate sheaths and in summer without. In the field, this hybrid is striking for its vegetative traits being close to *C.acuta*, but it has narrow and long female spikes (longer than those of *C.acuta*), especially the lowest one, which is pedunculate, interrupted at the base down to individual flowers and often pendent. The lowest bract sometimes exceeds the inflorescence, a character inherited from *C.acuta* (Koopman et al. 2018), but it is often shorter than, or as long as, the inflorescence. Carex×vratislaviensis is usually partially or fully fertile, less often sterile. In the field, backcrosses from the hybrid swarm are fertile and their traits match the variability of either parent. These plants are morphologically indistinguishable in the field from parental species. The only distinctive trait of this hybrid is the persistent small or larger red-brown scale-like basal sheaths.

#### Ecology.

Both parental species are relatively commonly found, most often in the floodplains of large rivers, where both find suitable habitats (*C.buekii*: gravel-sand terraces covered with clay and littoral embankments; *C.acuta*: oxbows, reservoirs, eutrophic wetlands in floodplains with nutrient-rich sediments) (Kaplan et al. 2018). Most localities of C.×vratislaviensis correspond with the distribution of *C.buekii*, however, it was also found on banks of lakes and in adjacent marshes where *C.acuta* usually grows (Koopman et al. 2018). Řepka (2023) recently described large populations of the hybrid on the banks of the River Elbe near the town of Děčín (northern Bohemia), and at the edge of the field, a unique habitat completely outside the requirements of both parental species.

#### Distribution.

It has been recorded so far in Austria, Czech Republic, Germany, Hungary, Italy, Poland, and Slovakia (Koopman 2022).

Carex×vratislaviensis is an independent hybridogenous taxon (nothospecies) living autonomously in nature, mostly fully or partially fertile, and spreads spontaneously in the landscape. In the Czech Republic, it is currently documented in approximately 400 extensive populations. Based on current knowledge, it is now the most abundant hybrid (nothospecies) of the genus *Carex* in the Czech Republic. It has an excellent ability of clonal reproduction, and its utricles are spread by water birds to other habitats. At some habitats, especially in older meadows in the floodplains of large rivers, it can strongly dominate over the parental species or grow completely independently without their presence. In our opinion it can be compared with the hybridogenous *C.recta* Boott, also from the section Phacocystis, which has originated from hybridisation between *C.aquatilis* Wahlenb. and *C.paleacea* Schreb. Ex Wahlenb. (Standley 1990). It is presumed that C.×vratislaviensis influences other species and hybrids by its gene flow and forms triple hybrids or at least simply affects their fertility in situ (and the subsequent formation of empty utricles and thus empty spikes); however, this process needs further research.

## Supplementary Material

XML Treatment for Carex × ligniciensis

XML Treatment for Carex × vratislaviensis

## ﻿Additional information

### Author ORCIDs

Helena Więcław https://orcid.org/0000-0002-8165-6532

Radomír Řepka https://orcid.org/0000-0002-9993-0042

Jacob Koopman https://orcid.org/0000-0002-8235-1108

### Data availability

All of the data that support the findings of this study are available in the main text.
